# Medial Septum Modulates Cellular Response Induced in Hippocampus on Microinjection of Cholinergic Agonists into Hypothalamic Lateral Supramammillary Nucleus

**DOI:** 10.3389/fnana.2017.00079

**Published:** 2017-09-15

**Authors:** Mohammed Z. Ariffin, Chian-Ming Low, Sanjay Khanna

**Affiliations:** ^1^Department of Physiology, Yong Loo Lin School of Medicine, National University of Singapore Singapore, Singapore; ^2^Department of Pharmacology, Yong Loo Lin School of Medicine, National University of Singapore Singapore, Singapore; ^3^Department of Anesthesia, Yong Loo Lin School of Medicine, National University of Singapore Singapore, Singapore; ^4^Neurobiology Program, Life Science Institute, National University of Singapore Singapore, Singapore

**Keywords:** c-Fos, cingulate cortex, carbachol, nicotine, lateral supramammillary nucleus

## Abstract

Cholinergic mechanisms in supramammillary nucleus (SuM), especially the lateral SuM (lSuM) modulates septo-hippocampal neural activity. The lSuM, as compared to the contiguous medial SuM (mSuM) has relatively dense projections to hippocampus and cingulate cortex (Cg). In the present study, we have investigated whether the effects of cholinergic activation of SuM on hippocampal and cortical neural activities involve a cooperative interaction with the medial septum (MS). Microinjection of the broad-spectrum cholinergic agonist, carbachol, or the cholinergic-nicotinic receptor agonist, nicotine, into the lSuM and the mSuM in urethane anesthetized rat evoked a similar pattern of hippocampal theta rhythm. Despite that, only the lSuM microinjections resulted in an increase in expression of c-Fos-like immunoreactivity (c-Fos-ir) in neurons, including interneurons, of the ipsilateral hippocampus with a very dense expression in dentate gyrus. Likewise, a robust induction of c-Fos-ir was also observed in the ipsilateral Cg. Inhibition of the MS with muscimol pre-treatment attenuated both carbachol-evoked c-Fos-ir and theta activation. The findings indicate that cholinergic–nicotinic mechanisms in lSuM evoke not only neural activation via the ascending synchronizing pathway but also an MS-modulated expression of the plasticity-related molecule c-Fos in cortical regions that are strongly innervated by the lSuM.

## Introduction

The septo-hippocampus (SH) and the different hypothalamic regions, including the supramammillary (SuM) region are linked anatomically and physiologically (Kirk and McNaughton, 1991, 1993; Kocsis and Vertes, 1994; Leranth and Kiss, 1996; Risold and Swanson, 1996; Vertes and Kocsis, 1997; Bland and Oddie, 1998; Jiang and Khanna, 2004; Pan and McNaughton, 2004). Notably, electrophysiological analysis suggests that the SuM and SH establish a bi-directional loop in mediation of theta wave activity, theta being a 3–12 Hz rhythmic slow activity that reflects synchronization of hippocampal neurons in processing of information (Kocsis, 2006; Kocsis and Kaminski, 2006).

Interestingly, the effect of SuM on hippocampal neuronal responses differs along its medio-lateral axis (Mizumori et al., 1989; Jiang and Khanna, 2006). Of note here is that locally applied cholinergic agonist preferentially recruits the lateral SuM (lSuM) such that lateral microinjections evoked a more robust and short latency effect on pyramidal cell excitability as compared to microinjections into the medial SuM (mSuM; Jiang and Khanna, 2006). Additionally, Ariffin et al. (2010) showed that, in anesthetized rat, nicotinic receptor mechanisms in the lSuM mediate the suppression of the CA1 population spike and the activation of hippocampal theta wave activity evoked on stimulation of reticularis pontis oralis (RPO) or on injection of the noxious agent, formalin, into a hind paw. Interestingly, RPO is part of the mesopontine tegmental anesthesia locus and inactivation of the region leads to impaired consciousness (Devor and Zalkind, 2001). Indeed, the afore-mentioned pattern of neural responses elicited in CA1 on RPO and sensory stimulation is associated with behavioral activation/arousal (Buzsáki, 1989, 2002; Khanna, 1997; Bland and Oddie, 2001; Ma et al., 2002; Hasselmo and McGaughy, 2004; Tai et al., 2006).

During the aroused state, the level of c-Fos, a transcription factor that is expressed in neurons on synaptic excitation, is relatively higher in hippocampal regions of rodents. This is observed during awake state as compared to sleep/anesthetized state, on exposure to novel environment and with mild stress (Pompeiano et al., 1994; Pace et al., 2005; Abulafia et al., 2009; Ariffin et al., 2013). Interestingly, the c-Fos protein affects excitability of hippocampal neurons and is also implicated in learning and memory (Guzowski, 2002; Zhang et al., 2002; Fleischmann et al., 2003).

Likewise, c-Fos immunohistochemistry suggests that SuM neurons are activated on exposure to novel environment and during REM hypersomnia which is marked by changes in cellular activity of hippocampus (Ito et al., 2009; Renouard et al., 2015; Billwiller et al., 2017). Conversely, inactivation of the SuM attenuates theta activation in parallel with decrement in animal arousal and behavioral activation (Ma and Leung, 2006, 2007).

In addition, it is notable that (a) medial septum (MS) modulates hippocampal theta activation induced on stimulation of SuM (Jiang and Khanna, 2004; Pan and McNaughton, 2004). Indeed, both SuM and MS comprise part of an ascending relay to hippocampus that is involved in hippocampal theta activation and (b) both the SuM, especially the lSuM, and MS project to the cingulate cortex (Cg; Saper, 1985). Thus, based on the foregoing and the evidence cited in the preceding text, we hypothesize that (a) the cholinergic activation of SuM, especially lSuM as compared to mSuM, evokes a robust expression of c-Fos in the hippocampus and the Cg, and (b) cortical c-Fos induced on cholinergic activation of SuM is modulated by MS. During the experiments we also monitored hippocampal theta wave activity evoked on SuM manipulations so as to compare and contrast the pattern of theta activations vis-a-vis the induction of c-Fos in these experiments.

## Materials and Methods

### Animals

Experiments were performed on male Sprague–Dawley rats (300–350 g). The experimental procedures were approved by the Institutional Animal Care and Use Committee (IACUC; protocol number R13-5855) at the National University of Singapore. A total of 30 animals were used in these experiments.

### Experimental Setup

As reported previously (Jiang and Khanna, 2004, 2006; Ariffin et al., 2010), the animals were anesthetized (urethane, 1 g/kg i.p., Sigma, United States), mounted onto a stereotaxic frame, and burr holes drilled on the exposed skull at the selected stereotactic coordinates to facilitate electrophysiological recording and chemical stimulation of the targeted brain regions.

A carbon fiber microelectrode (Armstrong-James and Millar, 1979) was lowered into the hippocampus at an angle of 15° to the vertical so as to target the left dorsal field CA1 (∼P3.0 mm from Bregma, ∼L2.0 mm from midline; Paxinos and Watson, 2009). Exmire microinjection syringes (Ito Corporation, Japan) were directed toward the left SuM nucleus (SuM; ∼P4.7 mm from Bregma, ∼L0.6–1.0 mm from midline, V8.7 mm from cortical surface; Paxinos and Watson, 2009) and the MS (∼A0.5 mm from Bregma, ∼L0.0 mm from midline, and ∼V7.0 mm from cortical surface; Paxinos and Watson, 2009). The syringes were lowered vertically down. Once the electrodes and needles were in place, the animal was allowed to recover for at least 3 h before the start of the experiment. This was done so that level of c-Fos, which may increase during surgery and setting up, recovers back to the baseline (Dragunow and Hughes, 1993; Spencer and Houpt, 2001).

### Electrophysiological Recording

Hippocampal field activity (1–100 Hz) was amplified 500× (Grass amplifier; Astromed, Inc., United States), digitized at 256 Hz via the Power1401 AD converter, and captured on Spike2 program (Cambridge Electronic Design, United Kingdom). Data were analyzed offline. During electrode placement, the recording electrode was lowered in 10 μm steps till a maximum was reached in the size of the spontaneous theta wave activity. Such a maximum is observed around the stratum lacunosum-moleculare to fissure of the hippocampus.

### Drugs and Microinjections

Either carbachol (0.1 μl of 0.156 μg/μl; Sigma, United States; Jiang and Khanna, 2006) or nicotine tartrate (0.1 μl of 12 μg/μl; Sigma, United States; Ikemoto et al., 2006) was microinjected into the mSuM or the lSuM. Carbachol is a broad spectrum cholinergic agonist (Ariffin et al., 2010) while nicotine is an agonist at the nicotinic cholinergic receptors. Muscimol hydrobromide (0.5 μl of 2 μg/μl; Sigma, United States; Lee et al., 2011), a GABA mimetic, was microinjected into the MS. All drugs were dissolved in vehicle made of 0.5% w/v Alcian blue dye (Sigma, United States) solution in saline (VWR, Germany). As microinjection control, vehicle was microinjected into SuM.

Functionally, intra-SuM microinjection of carbachol at the selected concentration elicits robust hippocampal theta activation (Jiang and Khanna, 2006). The concentration of intra-SuM nicotine used in this study is known to result in reinforcing behavioral effects (Ikemoto et al., 2006). While intraseptal muscimol at the aforementioned concentration results in suppression of hippocampal theta activation induced on RPO stimulation (Lee et al., 2011).

### Experimental Protocol

**FIGURE 1:**
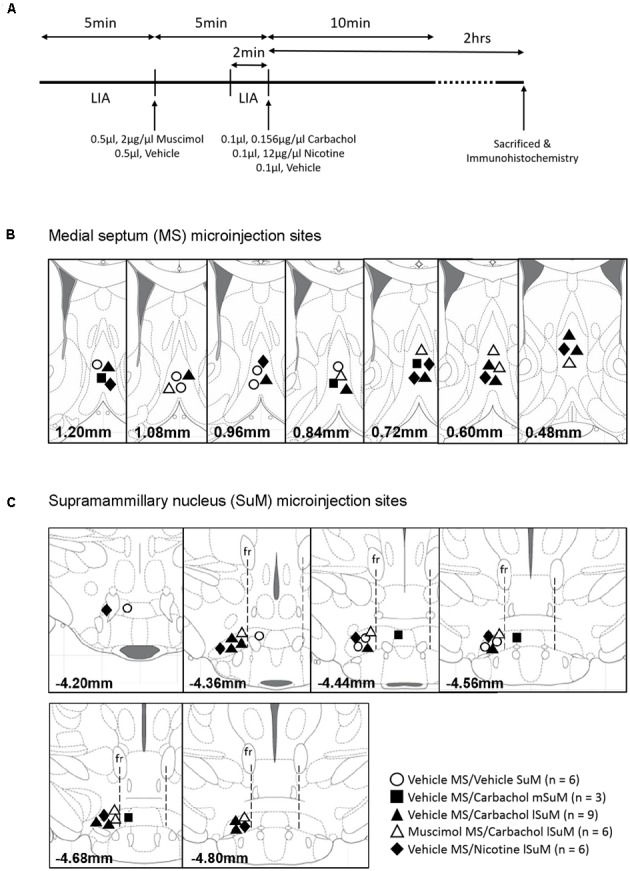
Timeline of the experiments and microinjection sites in the medial septum (MS) and supramammillary nucleus (SuM). **(A)** Experiments were carried out according to the timeline shown in the figure. After 5 min of large irregular field activity (LIA), muscimol (0.5 μl, 2 μg/μl) or the corresponding vehicle was microinjected into the MS. Hippocampal field activity was monitored for another 5 min after microinjection following which 0.1 μl of carbachol (0.156 μg/μl), nicotine (12 μg/μl), or the corresponding vehicle was microinjected into the medial SuM (mSuM) or the lateral SuM (lSuM). The field activity was monitored for another 10 min and the animals were sacrificed 2 h after microinjection into SuM. The animal brain was excised for immunohistochemistry. **(B,C)** Diagrammatic representations of the microinjection sites in the MS **(B)** and SuM **(C)**. The number at the bottom of each diagram represents the anterior–posterior coordinate of the diagram from the Bregma (Paxinos and Watson, 2009). The vertical line from fasciculus retroflexus (fr; **C**) divides the SuM into the lSuM or mSuM (Jiang and Khanna, 2006). Note that microinjections were directed only into the left lSuM. Microinjections of “Vehicle” into the mSuM and lSuM were combined as the “SuM” site.

In each experiment, a 2 min segment of spontaneous theta wave activity was recorded in the period preceding investigations involving drug microinjections. The spontaneous theta was used as a reference for later analysis (see Data Analysis). Drug microinjections were performed against the background of large irregular activity (LIA) in the hippocampal field activity. Investigations with drug microinjections involved the following protocol (**Figure 1A**): (a) monitoring 5 min of hippocampal field activity before and after microinjection of muscimol or vehicle into MS and (b) monitoring 10 min of hippocampal field activity after intra-SuM microinjection of one of the following drugs 5 min after intraseptal microinjection: carbachol, nicotine, or vehicle. The animal was sacrificed 2 h after the SuM microinjection (**Figure 1A**).

### Histology

#### Perfusion

Animal was deeply anesthetized with urethane (1.5 g/kg i.p.; Sigma, United States) and transcardially perfused with cold 0.05 M sodium nitrite (VWR, Germany) followed by 4% paraformaldehyde (Sigma, United States) in 0.1 M phosphate buffer (VWR, Germany). The brain was removed and kept overnight in the above fixative.

#### Sectioning

The brain was sectioned into 60 μm coronal sections using a vibratome (Leica VT1200 Semi-Automatic Vibrating Blade Microtome, Leica Microsystems, Germany). Every 50th section through the Cg (A2.30–P0.10 mm), and the anterior (P2.30–2.80 mm), middle (P3.14–3.80 mm), and posterior (P4.80–5.30 mm) hippocampus were collected for immunolabeling. Sections that contained the needle track and/or the dye spot were collected so as to identify the microinjection site.

#### Immunohistochemistry

All procedures were carried out at room temperature and with shaking unless otherwise indicated. The procedure used here for immunolabeling has been described before (Khanna et al., 2004; Lee et al., 2008; Ariffin et al., 2013). Briefly, endogenous catalase activity was abolished by incubating the sections in 0.03% v/v hydrogen peroxide (VWR, Germany) in 0.05 M Tris buffered saline (TBS; VWR, Germany) for 20 min. Sections were then washed with TBS and blocked with 3% w/v bovine serum albumin (BSA; Sigma, United States) in TBS with 0.1% v/v Triton (VWR, Germany) for 2 h. Sections were subsequently incubated with rabbit anti-c-Fos primary antibody (1:2500, Cell Signaling, United States) for 70 h at 4°C, followed by overnight incubation with biotinylated goat anti-rabbit secondary antibody (1:1000; Sigma, United States). All antibodies were diluted in the blocking medium. Subsequent to incubation with the antibodies, the sections were incubated with avidin–biotin–peroxidase complex solution (ABC, VectaStain Elite; Vector Laboratories, United States) for 3 h followed by treatment with 0.1% w/v diaminobenzidine (Sigma, United States). Once the chromogen had developed, the reaction was quenched by transferring the sections into TBS. Subsequently, sections were mounted on gelatin-coated slides, air dried, dehydrated with ethanol and xylene, and cover slipped with Micromount mounting medium (Leica, Germany).

#### Immunofluorescence

In some experiments, two sections each from the anterior, middle, and posterior hippocampus were processed for fluorescence labeling. Sections were blocked in 3% w/v BSA in TBS solution and incubated for 70 h at 4°C with rabbit anti-c-Fos primary antibody (1:2500, Cell Signaling, United States) together with one of the following antibodies – (i) mouse anti-parvalbumin (PV; 1:2000; Sigma, United States), (ii) mouse anti-somatostatin (SOM; 1:25; GeneTex Inc., United States), or (iii) mouse anti-neuronal nitric oxide synthase (nNOS; 1:50, Santa Cruz Biotechnology, United States). PV, SOM, and nNOS are markers of hippocampal interneurons (Freund and Buzsáki, 1996). After washing with TBS with 0.1% v/v Triton (TBS-T; 5 min × 3), sections were incubated with Alexa Fluor 488 tagged donkey anti-rabbit secondary antibody and Alexa Fluor 647 tagged donkey anti-mouse secondary antibody (1:400; Molecular Probes, United States) for 1 h at room temperature in the dark. Sections were then washed with TBS-T (5 min × 3), mounted on gelatin-coated slides, and cover slipped with Gelmount (Sigma, United States) aqueous mounting medium. Sections were kept in the dark at 4°C before imaging.

#### Nissl Stain

Sections containing the needle track and/or dye spot were air-dried on gelatin-coated slides and incubated in 0.5% w/v cresyl violet stain. Sections were sequentially dehydrated and rehydrated in increasing concentrations of ethanol (VWR, Germany) and xylene (VWR, Germany), and cover slipped with Micromount mounting medium (Leica, Germany).

### Confocal Microscopy

Fluorescent images were captured on the Zeiss LSM510 confocal microscope (Carl Zeiss, Germany) that was equipped with an Argon laser of 488 nm wavelength and a combination of Helium–Neon laser of 543 and 633 nm wavelengths, respectively. Images of Alexa Fluor 488 fluorescence were viewed with a 505–530 nm band pass filter, while Alexa Fluor 647 fluorescence was viewed with a 650 nm low pass filter. Images were captured with a pinhole of 1.0 Airy units at 20× magnification.

### Data Analysis

#### Quantification of Immunolabeling

c-Fos-like immunoreactive (c-Fos-ir) cells from the following regions were counted bilaterally: (a) hilus of the dentate gyrus (DG) and (b) the different layers of fields, CA1, CA2, and CA3, namely stratum oriens, stratum pyramidale, stratum radiatum, and stratum lacunosum-moleculare. c-Fos-ir in the granule layer of the DG was quantified by measuring the density of staining. The numbers of c-Fos-ir cells in the Cg were counted for the regions Cg1 and Cg2. Cellular layers of the hippocampus and cingulate regions were demarcated according to Paxinos and Watson (2009).

Sections were digitized at 750 dpi at 4× magnification (Nikon Eclipse, Japan) using the Montage Explorer software (Synoptics, United Kingdom). Positively immunolabeled neurons were quantified using the MCID software (Imaging Research Inc., United Kingdom). As previously (Ariffin et al., 2013), the criteria for identification of c-Fos-ir neurons were (i) label intensity being 3× of the background, (ii) area of label being >10 pixel, and (iii) form factor of label being >0.8. Form factor reflects the roundness of the labeled neuron. A form factor of 1.0 indicates a perfectly round structure. The background intensity was measured from label-free region of the sections. The regions of interest were demarcated using MCID’s free-form outline function.

However, the relative optical density (ROD) was used to measure the density of labeling in the granule cell layer of the DG. This method was followed since the DG granular layer was observed as tightly packed with immunopositive neurons following drug microinjections into lSuM. The ROD was computed by quantifying the gray level intensity per pixel. The number of pixels was determined by the size of the demarcated area. A higher ROD level indicates a denser immunolabeling.

For each experiment, the total number of c-Fos-ir neurons in each region was summed and averaged across the number of sections analyzed to determine the average number of FLI neurons per section. c-Fos-ir labeling of the DG granular layer was analyzed by calculating the ratio of the ROD level of the ipsilateral (to microinjection side, i.e., left) to the contralateral (to the microinjection side, i.e., right) granule cell layer. The average number of cFos-ir neurons and ratio of labeling intensity was subsequently averaged across the experimental group. Eight to twelve hippocampal sections and 6–10 sections across the Cg were analyzed per animal.

#### Qualitative Analysis of Double Labeled Immunofluorescence Labeling

c-Fos is a nuclear protein and was observed as a green fluorescent spherical structure. PV, SOM, and nNOS are cytoplasmic proteins and observed as red fluorescent which fills the soma and neuritic extensions of the neuron. Neurons double labeled for c-Fos-ir and one of the interneuronal markers were observed as a green structure surrounded by red immunofluorescent labeling. For some neurons, there is an overlap of the cytoplasm and the nucleus. In this case, the double labeled neurons were observed as a yellow structure surrounded by red immunofluorescent labeling.

#### Analysis of Hippocampal Field Activity

Hippocampal field activity was analyzed offline. Raw data were first low pass filtered at 40 Hz using the Finite Impulse Response (FIR) filter of the Spike2 software. Latency to theta, and theta duration, power, and frequency of the filtered waveform were analyzed in 1 min blocks post-SuM microinjection. Theta duration was the duration, in seconds per minute (s/min), of 3–6 Hz theta rhythmic oscillations in the hippocampal field activity trace. Latency to theta (s) was the time to onset of continuous theta in the first minute following SuM microinjection.

Theta peak power and frequency were calculated using Fast Fourier Transform (FFT; resolution of 0.5 Hz). The FFT analysis was performed on at least 2 s artifact free segments of theta field activity segments to compute power (FFT theta peak power; mV^2^) and frequency (FFT theta peak frequency; hertz). Note that the FFT theta peak power was expressed as the “relative theta power” which is the ratio of FFT theta peak power observed after microinjection into SuM to the FFT theta peak power of 2 min segment of spontaneous theta wave activity.

The peak effect of intra-SuM carbachol on hippocampal theta response has been shown to occur within the first 2 min following microinjection (Jiang and Khanna, 2006). Thus, theta duration, peak FFT frequency, and peak theta power were averaged across the first and second 1 min blocks.

### Statistical Analysis

Data were expressed as mean ± SEM. Comparisons between three or more groups were made using Kruskal–Wallis statistic followed by Dunn’s multiple comparison test. While the two-tailed Mann–Whitney non-parametric unpaired *t*-test was used for comparison between two groups. Significant difference was accepted at *p* < 0.05.

## Results

### Forebrain c-Fos Like Immunoreactivity Elicited by Microinjection into SuM

In these experiments, animals were microinjected with combination of muscimol (or the corresponding vehicle) and a cholinergic agonist (or the corresponding vehicle) into MS and SuM, respectively (**Figures 1B,C**). Hippocampal field activity was also recorded from these animals. The analyses of field activity are reported in the “Effect of Intra-MS Muscimol on SuM Driven Hippocampal Theta and the Relationship to c-Fos-ir Induction” section.

**FIGURE 2:**
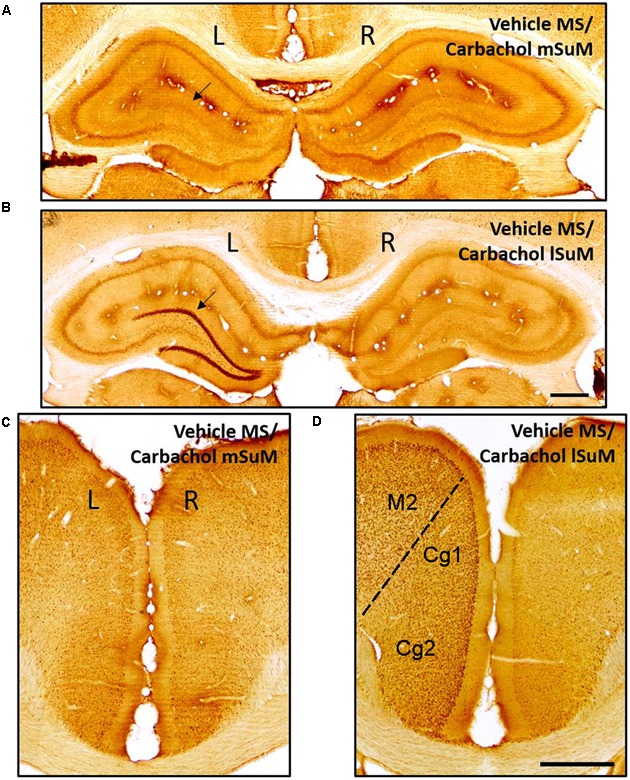
c-Fos protein is induced in the hippocampus and cingulate cortex (Cg) on microinjection of carbachol into the lSuM nucleus. Digital images showing c-Fos-like immunoreactivity (c-Fos-ir) in coronal sections taken through the hippocampus (**A,B**; posterior ∼2.76 mm from Bregma) and the Cg (Cg1 and Cg2; **C,D**; anterior ∼2.28 mm from Bregma). c-Fos-ir neurons appear as darkly stained against the background. Note that microinjection of carbachol into left lSuM (**B,D**; see **Figure 1**) but not the left mSuM (**A,C**; see **Figure 1**) induced c-Fos-ir on the ipsilateral (left, L on figure) but not the contralateral (right, R on the figure) hippocampus and Cg. The c-Fos-ir is particularly evident in the granule cell layer of the dentate gyrus (arrow in **B**) and Cg at the selected magnification (scale bar represents 100 μm). Besides Cg, an induction of c-Fos is also observed in other regions of the ipsilateral cortex such as the secondary motor cortex (M2), which is shown in **(D)**.

Brain tissue sections containing the Cg and hippocampus (anterior, middle, and posterior segments) were immunolabeled for c-Fos-ir. The immunolabeled neurons were identified as darkly stained against a lighter brown background as previously described (Khanna et al., 2004; Ariffin et al., 2013). Visual inspection under microscope indicated that the expression of c-Fos-ir was relatively robust on microinjection of carbachol into lSuM of animals’ microinjected with vehicle into MS (Vehicle MS/Carbachol lSuM; **Figure 2**). In comparison, little or no c-Fos-ir was induced on microinjection of carbachol into mSuM of animals’ microinjected with vehicle into MS (Vehicle MS/Carbachol mSuM; **Figure 2**). The expression of c-Fos-ir was dominantly ipsilateral and was observed across the different layers of the hippocampus and in the hilus, was most prominent in the DG granule cell layer and relatively marked in the Cg (**Figure 2**). Besides Cg, c-Fos induction was also observed unilaterally covering a broad area of the cortex, ipsilateral to the lSuM microinjections (e.g., see **Figure 2C**). Although c-Fos-ir was observed throughout the ipsilateral cortical mantle, analysis was restricted to the Cg region.

**FIGURE 3:**
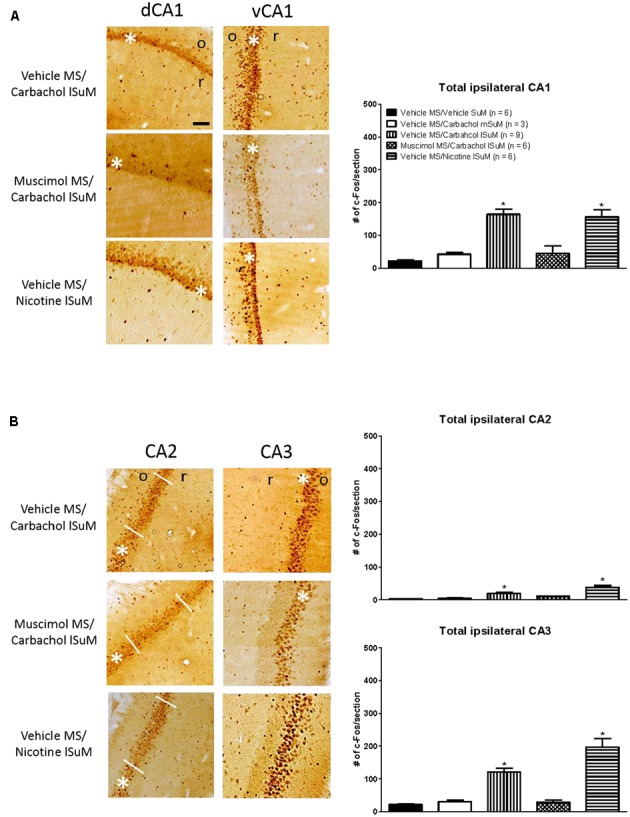
Medial septum modulates the carbachol-induced expression of c-Fos in the ipsilateral hippocampus. **(A)** On left are digital images showing c-Fos-like immunoreactivity (c-Fos-ir) in coronal sections taken through the dorsal hippocampus field CA1 (dCA1; posterior ∼3.00 mm from Bregma) and the ventral hippocampus field CA1 (vCA1; posterior ∼4.80 mm from Bregma). c-Fos-ir neurons are observed as a dark spherical structures against a lighter brown background. White asterisk on the figures indicates the pyramidal cell layer while “o” and “r” on the figures denote stratum oriens and stratum radiatum, respectively. The sections are taken from animals’ microinjected with combination of vehicle and a cholinergic agonist (carbachol or nicotine) into MS and SuM, respectively. The combination is denoted by “treatment region/treatment region” shown on left of the digital images. Thus, for example, an experiment involving microinjection of vehicle into MS and the cholinergic agonist, carbachol, into lSuM is indicated as “Vehicle MS/Carbachol lSuM”. On right is histogram illustrating changes in the number of c-Fos-ir neurons in CA1 on microinjection into MS and SuM. Note the robust increase in c-Fos-ir in the “Vehicle MS/Carbachol lSuM” and “Vehicle MS/Nicotine lSuM” groups. The carbachol-induced increase was prevented by microinjection of muscimol into MS (Muscimol MS/Carbachol lSuM). **(B)** Effect of drug microinjections on the number of c-Fos-ir neurons in hippocampal fields CA2 and CA3. The figure is built as explained for **(A)**. Data are mean ± SEM. Significant difference (*p* < 0.05): ^∗^ vs. Vehicle MS/Vehicle SuM and Vehicle MS/Carbachol mSuM. Statistical analyses were performed using Kruskal–Wallis statistic followed by Dunn’s Multiple Comparison Test. Scale bar represents 100 μm.

**FIGURE 4:**
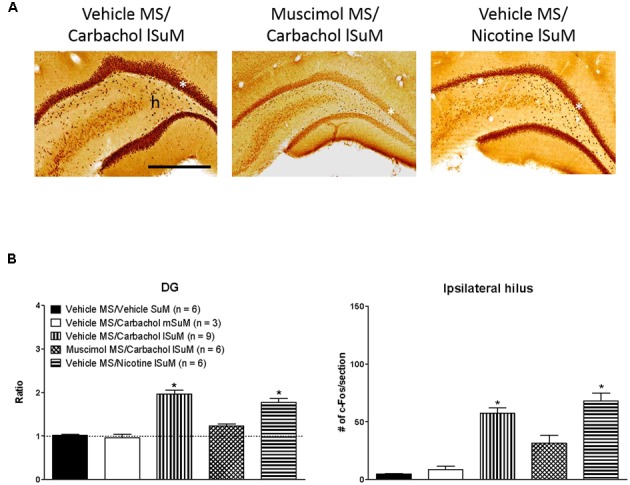
Medial septum modulates lSuM-induced expression of c-Fos-ir in the ipsilateral dentate gyrus (DG). **(A)** Digital images showing c-Fos-ir in coronal sections taken through the dorsal DG (posterior ∼3.00 mm from Bregma). White asterisk on the figures indicates the granule cell layer. Note that the granule cell layer is densely packed with c-Fos-ir in Vehicle MS/Carbachol lSuM and Vehicle MS/Nicotine lSuM groups, but is almost absent in the Muscimol MS/Carbachol lSuM group. A similar pattern of induction of c-Fos-ir is observed in the hilus (h). **(B)** Histograms illustrating changes in the level of c-Fos-ir in DG granule cell layer (DG on the figure) and ipsilateral hilus on microinjection into MS and SuM. c-Fos-ir in the granule cell layer was quantified by estimating the ROD since the labeling was very dense in the DG. The intensity of label on the ipsilateral side was expressed as the ratio of the ROD on the ipsilateral side to the contralateral side. A ratio of 1 (marked by horizontal dotted line) indicates comparable ROD on the ipsilateral and the contralateral side. The histograms are built as in **Figure 3**. Data are mean ± SEM. Significant difference (*p* < 0.05): ^∗^ vs. Vehicle MS/Vehicle SuM and Vehicle MS/Carbachol mSuM. Statistical analyses were performed using Kruskal–Wallis statistic followed by Dunn’s Multiple Comparison Test. Scale bar represents 1.0 mm.

**FIGURE 5:**
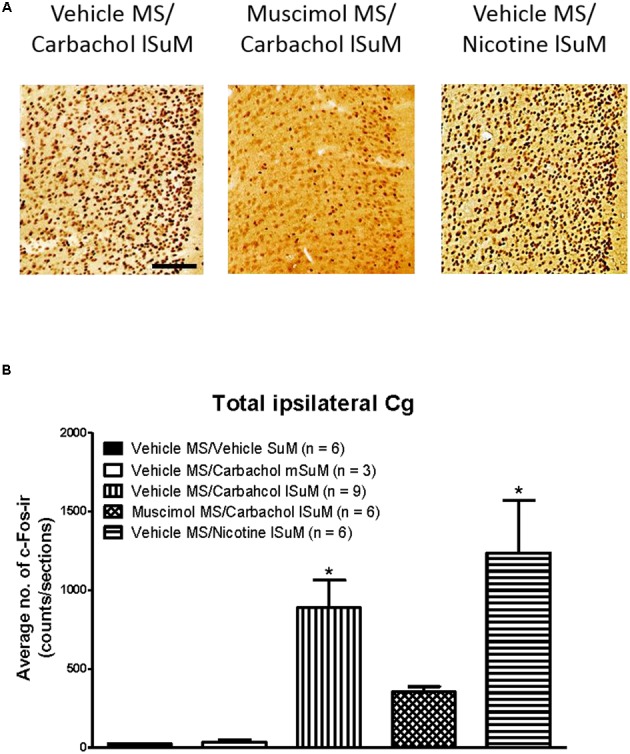
Medial septum modulates lSuM-induced expression of c-Fos-ir in the ipsilateral Cg. **(A)** Digital images showing c-Fos-ir in coronal sections taken through the Cg (anterior ∼1.80 mm from Bregma). Note the high level of c-Fos-ir in the Cg in Vehicle MS/Carbachol lSuM and Vehicle MS/Nicotine lSuM groups. The level of the label is relatively very low in the Muscimol MS/Carbachol lSuM group. **(B)** Histogram illustrating changes in the level of c-Fos-ir in ipsilateral Cg on microinjection into MS and SuM. The histograms are built as in **Figure 4**. Data are mean ± SEM. Significant difference (*p* < 0.05): ^∗^ vs. Vehicle MS/Vehicle SuM and Vehicle MS/Carbachol mSuM. Statistical analyses were performed using Kruskal–Wallis statistic followed by Dunn’s Multiple Comparison Test. Scale bar represents 100 μm.

Further, the c-Fos-ir was expressed throughout the length of the hippocampus. As an example, the expression of c-Fos-ir in the dorsal and ventral field CA1 is illustrated in **Figure 3**. The total counts reflecting c-Fos-ir along the septo-temporal length of each field of the hippocampus and hilus on carbachol or nicotine microinjection into SuM are illustrated in **Figures 3**, **4**. The ratio of ROD of the c-Fos-ir in DG is also illustrated in **Figure 4**, while the numbers of c-Fos-ir in the Cg (Cg1 plus Cg2) are illustrated in **Figure 5**.

Statistical analysis using Kruskal–Wallis statistics (*H*(4, 30) = 19.76 at least, *p* < 0.0006 at least) followed by Dunn’s Multiple Comparison Test indicated that compared to the values in the control “Vehicle MS/Vehicle SuM” group there was a statistically significant increase in the corresponding numbers of c-Fos-ir neurons in all regions of interest of the “Vehicle MS/Carbachol lSuM” and “Vehicle MS/Nicotine lSuM” groups, but not of the “Vehicle MS/Carbachol mSuM” group (**Figures 3**–**5**). On the other hand, prior microinjection of muscimol into MS attenuated the carbachol-induced increase such that the values representing the induction of c-Fos-ir neurons in the Muscimol “MS/Carbachol lSuM” group were low and, statistically, not different from the corresponding values in the control “Vehicle MS/Vehicle SuM” group (**Figures 3**–**5**).

Interestingly, microinjection of nicotine into lSuM (Vehicle MS/Nicotine lSuM group) tended to evoke a greater increase in c-Fos-ir in hippocampal fields CA2 and CA3 as compared to the “Vehicle MS/Carbachol lSuM” group (**Figures 3**, **4** and **Table 1**). Indeed, analysis of distribution of c-Fos-ir highlighted a trend toward higher level of c-Fos-ir in the “Vehicle MS/Nicotine lSuM” group, as compared to the “Vehicle MS/Carbachol lSuM” group, across all layers of the fields CA2 and field CA3, but not of field CA1 (**Table 1**). Particularly, it was noticeable that there was:

**Table 1:** Average number of c-Fos like immunoreactive (c-Fos-ir) neurons in hippocampal fields **(A)** CA1, **(B)** CA2, and **(C)** CA3.

	Str Or	Str Pyr	Str Rad	Str LM
**(A) c-Fos-ir in CA1**
Vehicle MS/Vehicle SuM	2.27 ± 0.44	3.82 ± 1.07	9.52 ± 1.31	6.52 ± 1.39
Vehicle MS/Carbachol mSuM	10.54 ± 5.48	10.50 ± 2.87	14.45 ± 0.45	6.72 ± 4.08
Vehicle MS/Carbachol lSuM	43.88 ± 4.94*	60.06 ± 4.58*	40.07 ± 7.54*	19.89 ± 4.84
Muscimol MS/Carbachol ISuM	16.71 ± 9.06	17.93 ± 10.03	8.40 ± 3.52	1.95 ± 0.38
Vehicle MS/Nicotine lSuM	43.23 ± 8.01*	51.06 ± 10.98*	42.12 ± 3.20*	19.18 ± 4.55
*H* statistic	*H*(4,30) = 19.44, *p* = 0.0006	*H*(4,30) = 19.38, *p* = 0.0007	*H*(4,30) = 21.97, *p* = 0.0002	*H*(4,30) = 16.23, *p* = 0.0027
**(B) c-Fos-ir in CA2**
Vehicle MS/Vehicle SuM	0.39 ± 0.22	0.77 ± 0.18	0.64 ± 0.17	0.57 ± 0.07
Vehicle MS/Carbachol mSuM	0.41 ± 0.36	1.24 ± 0.61	2.35 ± 0.92	None seen
Vehicle MS/Carbachol lSuM	4.96 ± 0.72*	5.59 ± 1.46	7.88 ± 1.41*	1.05 ± 0.50
Muscimol MS/Carbachol ISuM	3.83 ± 0.29	3.23 ± 0.40	2.01 ± 0.43	1.39 ± 0.36
Vehicle MS/Nicotine lSuM	8.88 ± 1.67*	11.66 ± 0.98*	10.92 ± 1.97*	6.71 ± 1.07*ˆ+
*H* statistic	*H*(4,30) = 21.50, *p* = 0.0003	*H*(4,30) = 21.28, *p* = 0.0003	*H*(4,30) = 21.81, *p* = 0.0002	*H*(3,27) = 15.34, *p* = 0.0015
**(C) c-Fos-ir in CA3**
Vehicle MS/Vehicle SuM	1.31 ± 0.58	9.26 ± 1.80	6.34 ± 0.90	4.11 ± 1.09
Vehicle MS/Carbachol mSuM	3.04 ± 1.30	10.76 ± 3.33	16.41 ± 3.16	None seen
Vehicle MS/Carbachol lSuM	18.47 ± 3.44*	48.20 ± 3.60	47.19 ± 6.40*	6.45 ± 2.26
Muscimol MS/Carbachol ISuM	4.33 ± 1.04	19.79 ± 9.61	7.58 ± 2.16	1.07 ± 0.59
Vehicle MS/Nicotine lSuM	40.36 ± 6.31*	64.90 ± 9.34*	75.29 ± 12.52*	15.75 ± 2.38
*H* statistic	*H*(4,30) = 24.52, *p* < 0.0001	*H*(4,30) = 18.08, *p* = 0.0012	*H*(4,30) = 23.97, *p* < 0.0001	*H*(3, 27) = 11.93, *p* = 0.0076

*The numbers of c-Fos-ir neurons in different layers of the hippocampus are shown on the table. The different layers are: stratum oriens (Str Or), stratum pyramidale (Str Pyr), stratum radiatum (Str Rad), and stratum lacunosum-moleculare (Str LM). Data are mean ± SEM. Statistical analysis was performed for each layer (i.e., along each column). Significant difference (*p* < 0.05 at least): ^∗^ vs. Vehicle MS/Vehicle SuM; + vs. Vehicle MS/Carbachol lSuM. Statistical analyses were performed using Kruskal–Wallis test with Dunn’s post hoctest*.

(a) A higher increase in c-Fos-ir in pyramidal cell layer of fields CA2 and CA3 on nicotine microinjection (**Table 1**). Indeed, the nicotine-induced increase was statistically significant as compared to the level seen on microinjection of vehicle in the control “Vehicle MS/Vehicle SuM” group. An increase was also seen on microinjection of carbachol into the lSuM in the “Vehicle MS/Carbachol lSuM” group which, however, tended not be as high as that seen on microinjection of nicotine and, also, was not statistically different from the level in the control “Vehicle MS/Vehicle SuM” group and
(b) A higher increase in c-Fos-ir in stratum-lacunosum-moleculare of fields CA2 and CA3 on nicotine microinjection (**Table 1**). Indeed, the nicotine-induced increase in field CA2 was statistically significant as compared to the level seen on microinjection of vehicle in the control “Vehicle MS/Vehicle SuM” group or on microinjection of carbachol into lSuM of the “Vehicle MS/Carbachol lSuM” group. A nicotine-induced increase was also seen in field CA3 which, however, did not reach significance. In contrast, the levels of c-Fos-ir in stratum lacunosum-moleculare of animals’ microinjected with carbachol in the “Vehicle MS/Carbachol lSuM” were low and comparable to the levels seen in the control “Vehicle MS/Vehicle SuM” group.

### Hippocampal Immunofluorescence Double Labeling

To identify the neurochemical nature of c-Fos-ir neurons in the hippocampus, selected sections were double labeled for c-Fos-ir and PV-, SOM-, or nNOS-ir. PV, SOM, and nNOS are markers of hippocampal interneurons (Freund and Buzsáki, 1996). Hippocampal fields CA1, CA3, and DG were examined.

**FIGURE 6:**
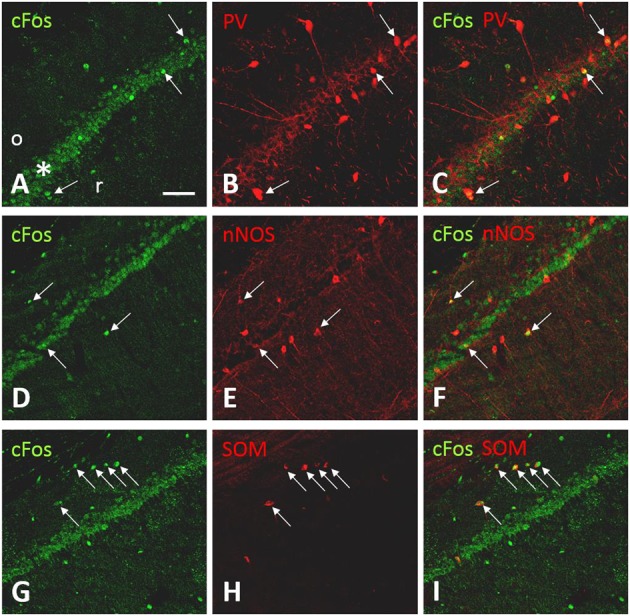
c-Fos-ir in CA1 is co-localized with markers of interneurons in CA1. Confocal fluorescent images showing the colocalization of c-Fos-ir and the interneuron markers parvalbumin (PV), neuronal nitric oxide synthase (nNOS), and somatostatin (SOM) in the field CA1 of the ipsilateral hippocampus following carbachol microinjection into the lSuM. c-Fos-ir was detected in the stratum pyramidale (^∗^), stratum oriens (o), and radiatum (r; **A,D,G)**. **(B**,**E**,**H)** illustrate the expression of PV-ir, nNOS-ir, and SOM-ir. Double-labeled neurons (arrows) were identified in stratum oriens, pyramidale, or radiatum **(C,F,I)**. Scale bar represents 100 μm.

In the CA1 (**Figure 6**), the pattern of c-Fos-ir was similar to that observed using the chromogen method. The soma of PV-ir neurons was found in mostly in stratum oriens, stratum pyramidale, and stratum radiatum of the CA1. Labeled neurites could be seen arising from the putative interneurons. Some neurites were seen entering the pyramidal cell layer (**Figure 6**). Double labeled cFos-ir/PV-ir neurons were found adjacent to or within the pyramidal cell layer (**Figures 6A–C**). While c-Fos-ir/nNOS-ir neurons were observed in the stratum oriens, stratum radiatum, and stratum pyramidale (**Figures 6D–F**). c-Fos-ir/SOM-ir neurons were detected only in the stratum oriens (**Figures 6G–I**). Likewise, a pattern of co-localization of c-Fos-ir with the three markers of interneurons was observed in the CA3 and hilus (data not shown).

### Effect of Intra-MS Muscimol on SuM-Driven Hippocampal Theta and the Relationship to c-Fos-ir Induction

**FIGURE 7:**
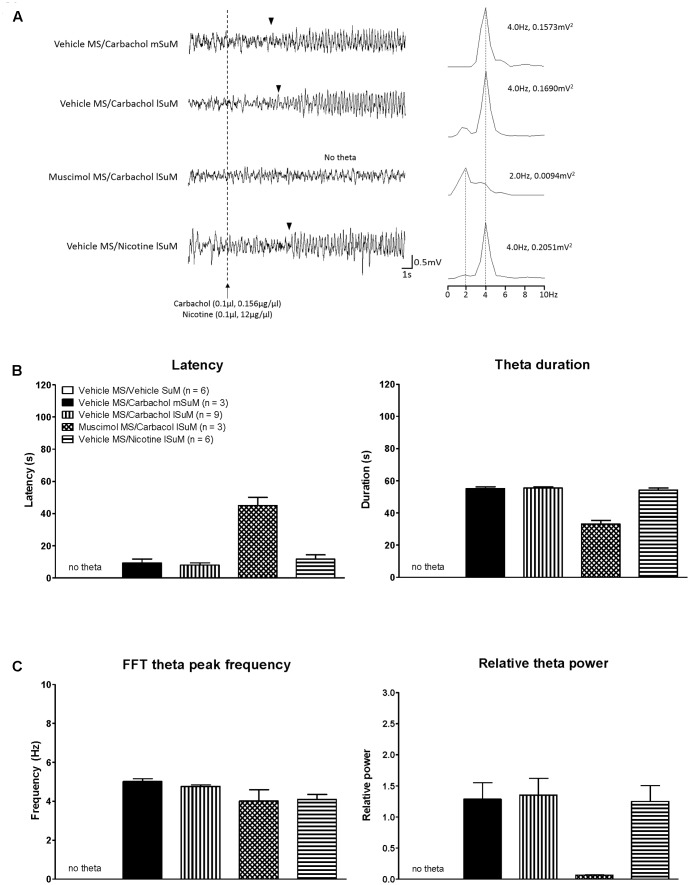
Medial septum modulates hippocampal theta wave activity induced on microinjection of cholinergic agonists into SuM nucleus. **(A)** Representative traces of hippocampal field activity recorded in anesthetized rat. Note the robust theta activation in animals microinjected with combination of vehicle into the MS and the cholinergic agonist, carbachol (0.1 μl, 0.156 μg/μl), into either the medial supramammillary nucleus (mSuM; Vehicle MS/Carbachol mSUM) or the lSuM (Vehicle MS/Carbachol lSuM). Likewise, robust theta activation was observed in the “Vehicle MS/Nicotine lSuM” animal where nicotine (0.1 μl, 12 μg/μl) was microinjected into lSuM instead of carbachol. Whereas microinjection of the GABA mimetic, muscimol (0.5 μl, 2 μg/μl), into MS blocked theta activation on subsequent microinjection of carbachol into lSuM (Muscimol MS/Carbachol lSuM). The cholinergic agonists (carbachol or nicotine) were microinjected 5 min after microinjection into MS. The vertical dotted line indicates the time of microinjection of carbachol or nicotine. The arrowheads indicate the onset of continuous theta wave activity after microinjection into SuM. The plot on right of each trace is fast Fourier transform (FFT) of 2 s of field activity from the onset of theta activity in the trace. In case of “Muscimol MS/Carbachol lSuM,” the FFT shown on right of the trace corresponds to the last 2 s of the trace. The value to right of FFT peaks, other than the FFT peak for “Muscimol MS/Carbachol lSuM” group, represents the FFT theta peak frequency and FFT theta peak power. The values for the “Muscimol MS/Carbachol lSuM” group represent the peak FFT values. **(B)** Histograms showing latency to onset of theta wave activity (left), duration of theta wave activity in different groups, and FFT parameters. The latency to theta was the time to onset of continuous theta wave activity following microinjection into SuM. The theta duration is the duration for which theta wave activity was observed after microinjection into SuM. Theta activation was observed in only three of six animals in the “Muscimol MS/Carbachol lSuM” group. Thus, the “*n*” value for this group was 3, instead of 6 for all theta parameters, including FFT theta peak frequency and the FFT theta peak power. **(C)** The FFT theta peak power is expressed as relative theta power. The “relative theta power” is the ratio of FFT theta peak power observed after microinjection into SuM to the FFT theta peak power of 2 min segment of spontaneous theta wave activity. Note that no theta wave activity was observed in “Vehicle MS/Vehicle SuM” group and thus this group is not represented by any data point. Data are mean ± SEM. Statistical analyses were performed using Kruskal–Wallis statistic followed by Dunn’s Multiple Comparison Test. Such analysis showed an overall significant effect of muscimol treatment on latency to theta, theta duration, and theta power, but not on theta frequency.

Theta wave activity was recorded from animals used for analyses of c-Fos (see the “Forebrain c-Fos Like Immunoreactivity Elicited by Microinjection into SuM” section). A robust hippocampal theta activation was observed in animals’ microinjected with combination of vehicle into MS and carbachol or nicotine into SuM, whether mSuM or lSuM (see the field activity traces in **Figure 7**). Indeed, carbachol- or nicotine-evoked hippocampal theta rhythm in these animals persisted throughout the 10 min of the recording period. The total duration of theta in the 10 min period on microinjection of carbachol into mSuM vs. lSuM was 590.3 ±_ 2.19 s (*n* = 3) vs. 591.0 *±* 1.28 s (*n* = 9) (two-tailed Mann–Whitney *t*-test, *U* = 13.50, *p* > 0.90, two-tailed).

Whereas microinjection of muscimol into MS strongly attenuated theta activation which was otherwise induced on microinjection of carbachol into lSuM (see the field activity traces in **Figure 7**). The theta wave activity induced in the first 2 min after SuM microinjection was subject to FFT analyses and the various FFT parameters were compared among the groups (**Figure 7**). Here, it is notable that no theta wave activity was observed in the first 2 min in three of six animals microinjected with carbachol in the “Muscimol MS/Carbachol lSuM” group. That is, in these three animals carbachol-induced theta activation was blocked with MS muscimol. As a result, only the theta parameters obtained from the remaining three animals could be used for the purpose of analyses (see next paragraph). Further, no theta was observed in the “Vehicle MS/Vehicle SuM” group. Thus, this group, although represented in **Figure 7**, has no data point.

Statistical analysis, using Kruskal–Wallis statistics also showed an overall significant effect of muscimol treatment on latency to theta, theta duration, and theta power (*H*(3,27) = 5.40 at least, *p* < 0.0021 at least), but not on theta frequency (*H*(3,27) = 0.017, *p* = 0.8969). While the muscimol-induced changes did not reach significance with the Dunn’s Multiple Comparison Test, the latency to theta activation was markedly increased, while theta duration and particularly the theta power were strongly attenuated as compared to the values in the “Vehicle MS/Carbachol mSuM,” “Vehicle MS/Carbachol lSuM,” and “Vehicle MS/Nicotine lSuM” groups. The values in the latter three groups were comparable. The preceding observations are consistent with a robust attenuation of lSuM carbachol-induced hippocampal theta activation by intraseptal muscimol.

Based on the foregoing analyses, the “Muscimol MS/Carbachol lSuM” group was demarcated into two sub-groups (**Table 2**), namely (a) one in which theta activation was attenuated but not blocked by intraseptal muscimol and (b) the other that showed a block of theta activation on microinjection of muscimol into MS. The effect on theta in the former presumably reflected an intraseptal muscimol-induced robust decrease, but not loss of septal neural activity while the change in the latter likely reflected a near loss of neural activity in the MS on microinjection of muscimol. The decrease in neural activity in the former is interpreted as robust since the power of theta wave was strongly attenuated with muscimol microinjection (**Figure 7C**).

**Table 2:** The total numbers of hippocampal c-Fos like immunoreactive (c-Fos ir) neurons in the different sub-field of the hippocampus and dentate gyrus (DG) in the “Muscimol MS/Carbachol lSuM” sub-groups.

	1	2	3	
	Vehicle MS/Carbachol lSuM (*n* = 9)	Muscimol MS/Carbachol lSuM (attenuation of theta activation) *n* = 3	Muscimol MS/Carbachol lSuM (block of theta activation) *n* = 3	*t*-test (2 vs. 3) *p*-value
Total CA1	163.90 ± 16.03	72.93 ± 42.59	17.06 ± 3.30	0.20
Total CA2	19.47 ± 3.38	11.40 ± 1.74	9.49 ± 0.22	0.40
Total CA3	120.30 ± 11.65	40.81 ± 6.59	14.73 ± 5.95	0.10
Total hilus	57.32 ± 4.87	46.12 ± 3.47	16.67 ± 3.39	0.10
DG ratio	1.96 ± 0.09	1.25 ± 0.07	1.21 ± 0.07	1.00

*The sub-groups were demarcated into two based on the efficacy of intraseptal (i.e., MS) muscimol in blocking hippocampal theta activation induced on microinjection of carbachol into lateral supramammillary nucleus (lSuM). Of the two sub-groups, theta activation was attenuated, but not blocked in one subgroup (see the column marked as 2), while theta was blocked in the other sub-group (see the column marked as 3). The numbers of c-Fos-ir neurons in the control “Vehicle MS/Carbachol lSuM” group are also shown for reference purpose (see the column marked as 1). Data are mean ± SEM. Statistical analyses were performed using two-tailed Mann–Whitney non-parametric unpaired *t*-test*.

A demarcation of the c-Fos-ir of the “Muscimol MS/Carbachol lSuM” group so as to parallel the separation of the group into sub-groups (a) and (b) showed that the numbers of c-Fos-ir neurons were low in both the sub-groups as compared to the values in the control “Vehicle MS/Carbachol lSuM” group (**Table 2**). Although statistically insignificant, the levels of c-Fos-ir tended to be higher in fields CA1, CA3, and hilus of the sub-group (a) as compared to (b) (**Table 2**). This suggests the possibility that the attenuation of the induction of c-Fos-ir in the “Muscimol MS/Carbachol lSuM” group was influenced, at least in part, by the strength of attenuation of septal neural activity by intraseptal muscimol.

## Discussion

Following microinjection of carbachol or nicotine into the lSuM, c-Fos-ir was induced in the ipsilateral hippocampus and Cg. The expression of c-Fos-ir was observed throughout the medial–lateral and septo-temporal axis of the hippocampus. The labeling was most dense in the DG granule cell layer where the labeling of the tightly packed granule cells was observed as a dark band. Labeled interneurons were also observed in the region. Furthermore, the expression of c-Fos-ir involved a cooperative interaction between lSuM and MS such that inactivation of MS prior to microinjection of carbachol into lSuM strongly attenuated the expression of c-Fos-ir in the DG, including the granule cell layer. The physiological observations have a basis in neuroanatomy wherein a robust projection is observed from the lSuM to both MS and the DG thus providing an anatomical route for lSuM to influence DG directly and also indirectly through MS (Haglund et al., 1984; Vertes, 1992; Vertes and McKenna, 2000). Indeed, an MS-dependent, robust increase in c-Fos-ir was also induced in the Cg on microinjection of carbachol into lSuM, the Cg being innervated by projections both from the lSuM and the MS (Saper, 1985; Vertes, 1992). Collectively, the foregoing suggests that lSuM and MS function cooperatively in modulating responses in cortical areas that receive overlapping innervation from the two regions.

Conversely, carbachol microinjection into mSuM that has relatively sparse projection to DG and virtually none to Cg (Vertes, 1992) failed to evoke a significant increase in c-Fos-ir in the two regions, even though the mSuM projects to MS. Despite the difference in the potential to induce c-Fos, the pattern of hippocampal theta activation on carbachol microinjection into lSuM and mSuM was quite similar. The SuM and MS comprise part of the ascending theta-synchronizing system affecting the hippocampal formation. This strengthens the current idea that cooperative interaction between SuM and MS projections to DG, and not neural activation alone via the ascending synchronizing system underpinned the molecular plasticity.

Indeed, the ability to induce c-Fos-ir on carbachol microinjection into lSuM declined in fields CA2–CA3 which receive relative moderate innervation from lSuM. The labeled neurons in the pyramidal layer of CA2 and CA3 were interspersed with non-labeled cells. Labeled interneurons were also observed. Intriguingly, c-Fos-ir was also induced in the pyramidal cell layer of field CA1 and in the local interneurons. However, lSuM does not project to hippocampal field CA1 and thus the induction of c-Fos in this region may mostly be a measure of the network activation and not reflect a direct excitatory modulation by lSuM efferent. In this regard, intra-hippocampal associational connections form a robust sequential excitatory relay from DG granule cells to field CA3 pyramidal cell and from field CA3 to field CA1 pyramidal cells. Such a relay might in part underpin the induction of c-Fos-ir field CA1.

Furthermore, as with DG, the labeling in the hippocampal fields was MS dependent. Notably, the numbers of lSuM carbachol-induced c-Fos-ir neurons expressed in the hippocampal fields, especially CA1 and CA3 as also the hilus, tended to be influenced by the strength of MS inactivation such that a stronger MS inactivation led to a lower expression of c-Fos-ir in the above-mentioned regions. Interestingly, the cholinergic activation of lSuM modulates the intra-hippocampal flow of information which may also negatively affect the ability to induce c-Fos-ir on cholinergic activation of lSuM. For example, electrophysiological data suggest that activation of lSuM inhibits the excitatory synaptic transmission via the Schafer collateral from CA3 pyramidal cells to CA1 pyramidal cells (Jiang and Khanna, 2004, 2006; Ariffin et al., 2010). Furthermore, *in vitro* and *in vivo* data suggest a high failure rate of excitatory synaptic transmission between dentate granule cell–CA3 pyramidal cells synapse, especially at the low firing rate of dentate granule cells (Henze et al., 2002; Mori et al., 2004). Potentially, therefore, the activation of lSuM together with physiological characteristics of hippocampal synapses prevents a widespread excitation of pyramidal cells via the intra-hippocampal associational pathway and thus dampens the expression of c-Fos-ir in hippocampal fields so that it is not commensurate with the robust expression of c-Fos-ir in granule cells. Strikingly, SuM-mediated sequential expression of c-Fos-ir in DG granule cells and hippocampal principal neurons occur potently under circumstance of intense neuronal excitation, such as seen during epileptic spiking (Saji et al., 2000). Such spiking, however, is not seen with cholinergic stimulation of SuM in the present study.

The similar pattern of effect of carbachol and nicotine on expression of c-Fos-ir in the different regions of interest suggests that nicotinic receptors in lSuM mediate the response to drug microinjections, at least in part. Indeed, pharmacological investigations indicate that carbachol at the concentration used in the current study has a mixed agonist like effect in SuM. In this regard, the effects of carbachol microinjected into SuM are antagonized by local administration of the muscarinic antagonist, atropine, or the nicotinic receptor antagonist, mecamylamine (Ariffin et al., 2010). Furthermore, both muscarinic and nicotinic receptors are observed in the SuM (Rotter et al., 1979; Wada et al., 1989; Le Novère et al., 1996), while the enzymes associated with acetylcholine synthesis and metabolism are also observed in the neurons of the region (Parent and Butcher, 1976; Tago et al., 1987; Lauterborn et al., 1993).

While mostly complementary, the effect of carbachol and nicotine also differed in that microinjection of nicotine, but not carbachol, induced c-Fos-ir in stratum lacunosum-moleculare of hippocampal field CA3 and CA2. Overall, the nicotine-induced expression of c-Fos-ir tended to be higher as compared to the expression induced with carbachol. However, a trend toward additional increase with nicotine microinjection was not observed in areas with high c-Fos-ir following carbachol microinjection. The latter include DG, where a very dense c-Fos-ir is induced in granule cell layer, and Cg. Further, the nicotine-induced increase was not observed in field CA1 which receives little or no direct input from lSuM. However, it is notable that we have not compared the dose relationship of the two agonists. Indeed, a parsimonious explanation is that the selected dose of nicotine is somewhat more efficacious and evokes a greater spatial spread of c-Fos-ir, preferentially into areas innervated by lSuM, such as fields CA2 and CA3 where the response to cholinergic activation in the present study is relatively sub-maximal.

Functionally, the role of lSuM-MS cooperative interaction in driving or modulating hippocampal and cortical plasticity and function remains unclear, though the lSuM is known to drive hippocampal induction of c-Fos during REM sleep hypersomnia (Renouard et al., 2015). Interestingly, a prominent source of cholinergic input to the lSuM is the MS region. Indeed, the majority of projection neurons from the MS to the lSuM are cholinergic in nature and the projection is relatively robust (Gonzalo-Ruiz et al., 1999). In turn, the lSuM projects to MS. The lSuM-MS projection is suggested to be mainly glutamatergic, although recent findings suggest that the rostral going projection from lSuM, especially to DG, may display a dual phenotype, localizing both glutamate and GABA as a transmitter (Kiss et al., 2000; Boulland et al., 2009; Soussi et al., 2010). While we have not directly examined the activity of the septal cholinergic neurons on carbachol-induced activation of lSuM, the induction of hippocampal theta on cholinergic activation of lSuM suggests that the septal cholinergic neurons are activated, especially since hippocampal theta wave activity in urethane anesthetized animals is strongly modulated by these neurons (Bland, 1986; Dannenberg et al., 2015). Furthermore, directed transfer function analysis of electrophysiological data suggests that the SuM and SH establish a bi-directional loop in mediation of theta wave activity (Kocsis, 2006; Kocsis and Kaminski, 2006). Indeed, relevant here is that sensory- and reticular stimulation-evoked hippocampal theta activity which is mediated via SuM is antagonized by cholinergic antagonist microinjected into lSuM (Ariffin et al., 2010). Similarly, in context of Cg, we have not directly explored the septal–Cg or hippocampal–Cg interactions in the present study. Nonetheless, published evidence indicates that the septal cholinergic neurons also modulate theta-related neural activity in the Cg (Borst et al., 1987). Put together, the preceding raises a possibility that the cholinergic mechanisms in lSuM are part of a bi-directional excitatory loop such that excitation of lSuM, as with microinjection of cholinergic agonist, excites MS cholinergic neurons which, on the one hand modulate hippocampal and cingulate neural activity including hippocampal theta activation, and, on the other hand, be part of excitatory feedback to lSuM. The feedback to lSuM may reinforce the output from the lSuM, including to the hippocampus and Cg where they either drive or interact with other input to affect hippocampal plasticity.

Also of potential functional interest is the qualitative observations that the SuM–MS axis influenced plasticity in a range of cortical regions. Thus, microinjection of the cholinergic agonists into the lSuM evoked c-Fos in a broad area of the ipsilateral cortex. In line with a cooperative interaction between lSuM and MS, the induction of cortical c-Fos on cholinergic activation of lSuM was prevented by inactivation of the MS region. Indeed, both the lSuM and septal neurons project to a range of cortical regions which, besides Cg, include the entorhinal cortex, the frontal cortex including the region of motor cortex, and the occipital cortex (Gaykema et al., 1990; Vertes, 1992). Moreover, the cortical projection from the MS region is much more extensive than that from lSuM (Gaykema et al., 1990). Perhaps, the activation of lSuM induces c-Fos in a wider swath of cortex than expected from lSuM–cortical projection alone partly because of activation of MS projections to the cortex. Furthermore, cortical regions such as entorhinal cortex that receive MS and lSuM projections have extensive connection with other cortical regions (Agster and Burwell, 2009). Potentially, such intra-cortical connection may modulate expression of c-Fos.

In summary, the present study shows that cholinergic–nicotinic activation of lSuM cooperatively influences with MS the induction cortical c-Fos, a plasticity-related molecule, which is consistent with the notion that the two regions form a functional network with the hippocampal formation and Cg.

## Author Contributions

Experiments were conceptualized by SK and carried out by MA; data were analyzed by MA; manuscript was written by MA and SK with inputs from C-ML.

## Conflict of Interest Statement

The authors declare that the research was conducted in the absence of any commercial or financial relationships that could be construed as a potential conflict of interest.
